# Genome-Wide Analysis of *WOX* Multigene Family in Sunflower (*Helianthus annuus* L.)

**DOI:** 10.3390/ijms24043352

**Published:** 2023-02-08

**Authors:** Ettore Riccucci, Cosimo Vanni, Alberto Vangelisti, Marco Fambrini, Tommaso Giordani, Andrea Cavallini, Flavia Mascagni, Claudio Pugliesi

**Affiliations:** 1 Department of Agriculture Food and Environment, University of Pisa, Via del Borghetto 80, 56124 Pisa, Italy; 2 Scuola Superiore Sant’Anna, Piazza Martiri della Libertà 33, 56127 Pisa, Italy

**Keywords:** *WOX* transcription factor family, *Helianthus annuus*, expression analysis, embryo development, ovule, inflorescence meristems

## Abstract

The WUSCHEL-related homeobox (*WOX*) is a family of specific transcription factors involved in plant development and response to stress, characterized by the presence of a homeodomain. This study represents the first comprehensive characterization of the *WOX* family in a member of the Asteraceae family, the sunflower (*H. annuus* L.). Overall, we identified 18 putative *HaWOX* genes divided by phylogenetic analysis in three major clades (i.e., ancient, intermediate, and WUS). These genes showed conserved structural and functional motifs. Moreover, *HaWOX* has homogeneously distributed on *H. annuus* chromosomes. In particular, 10 genes originated after whole segment duplication events, underpinning a possible evolution of this family along with the sunflower genome. In addition, gene expression analysis evidenced a specific pattern of regulation of the putative 18 *HaWOX* during embryo growth and in ovule and inflorescence meristem differentiation, suggesting a pivotal role for this multigenic family in sunflower development. The results obtained in this work improved the understanding of the *WOX* multigenic family, providing a resource for future study on functional analysis in an economically valuable species such as sunflower.

## 1. Introduction

*Homeobox* genes have been found in animals, fungi, plants, and many single-celled eukaryotes [1,2,3,4,5,6,7,8,9]. Likely, the first *homeobox* genes that could have appeared during early eukaryote evolution were probably derived from a helix–turn–helix (HLH) transcription factor (TF) [9]. Gene codifying for homeobox proteins contains a widely conserved domain of 180 bp, which encodes for a globular homeodomain (HB) of about 60 amino acids usually involved in DNA binding [8,9,10].

The first example of a homeotic mutation, named *bithorax* (*bx*), was uncovered in 1915 by Calvin Bridges in *Drosophila melanogaster* [10]. Since then, the number of studies on homeotic gene mutations has increased [11]. Nuclear genomes of invertebrate and vertebrate animals host homeotic genes, which encode for TFs that regulate gene expression and appear as tight collinear clusters of several loci (derived through serial gene duplications and divergence from a common ancestor) [12,13].

In plants, the first *homeobox* gene was identified in the early 1990s. Vollbrecht et al. [14] resumed the study of an old maize mutant named *Knotted-1* (*Kn-1*) in which clusters of cells located along lateral leaf veins continued to divide, forming characteristic growths known as knots [15].

*Homeobox* genes regulate numerous aspects of plant development. For example, maintenance of stem cells [16,17], hypogeal and epigeal growth [18,19], determination and symmetry of the floral organs [20,21], and response to biotic and abiotic stresses [22,23].

In particular, the *WUSCHEL* (*WUS*)*-*related *homeobox* (*WOX*) genes encode for a class of plant-specific homeobox TFs, which perform several key functions in plant development processes, such as organization and embryonic development, maintenance of stem cells, formation of various organs, and induction of somatic embryos [19,24,25]. These functions may be related to the increase in cell division rate and the prevention of early cell differentiation. Genetic analysis in *Arabidopsis thaliana* suggests a central role for the prototypic WOX-family member *WUS* gene in regulating stem cell fate throughout development. The *wus* mutants fail to organize a shoot meristem in the embryo properly. Post-embryonically, defective shoot meristems are initiated repetitively but terminate prematurely in aberrant flat structures [24]. In addition, *wus* floral meristems terminate prematurely in a central stamen [24]. In *A. thaliana*, 15 different *WOX* genes were identified. The specific function of each *WOX* gene is determined by spatiotemporal expression pattern and protein–protein interaction [25]. According to the phylogenetic analysis among WOX families of several species, these TFs are divided into three separate clades: WUS, intermediate, and ancient clades [26].

Within WOX TFs, the helix–loop–helix–turn–helix (HLHTH) secondary structure is characterized by α-helices, joined by a short loop and a short turn [27,28,29]. The second helix binds DNA through hydrogen bonds and hydrophobic interactions, in particular specific regions of the protein chains, establishing a bond with the exposed bases and with thymine methyl groups within the major DNA groove [25].

Members of the WUS clade also contain the WUS-box motif T–LX–LFPXX (where T stands for threonine, L for leucine, F for phenylalanine, P for proline and X for any amino acid) in the carboxyl-terminal region that distinguishes them from other homeobox TFs [26]. The WUS-box motif is essential for specific functions such as regulating the stem cell population of the shoot apical meristem (SAM) and controlling flower development [26,30,31]. In particular, members of the WUS clade possess two conserved amino acids (threonine and leucine) at the beginning of the WUS box, while WOX proteins belonging to the other clades show amino acid variations in the initial position.

Some WOX proteins also show a carboxyl-terminal ERF-associated amphiphilic repression (EAR) domain between the HB and the WUS-box that could act as an activation domain [25]. The EAR-like motif (LXLXL or LELXL) is commonly involved in transcriptional repression [30]. In *Arabidopsis*, a more complex EAR motif structure was identified, [LVI]–X–[LVI]–X–[LVI] (where V represents valine). This motif has been detected for all three major WOX clades [31]. In *Arabidopsis*, the C-terminal EAR domain in WUS, WOX5 and WOX7 regulates the repression activity in vegetative and floral meristems [31]; this can be partially mediated by interaction with other proteins such as TOPLESS (TPL), which interacts with WUS through the EAR motif [30,31,32]. In *Oryza sativa*, WOX11 and WOX3 do not possess the EAR motif; nevertheless, they can act as repressor [31,33,34]. In addition, a simple L–X–L motif has been detected in all *Arabidopsis* WOX proteins except for WOX8 and WOX10 [26].

Concerning specific functions of *WOX* genes, in *Arabidopsis*, the *WUS* and *WOX5* genes are involved in the maintenance of stem cell functioning in SAM cells and root apical meristem (RAM), respectively [35]. *WOX5* is also expressed in the early stages of lateral roots and cotyledon development [36]. In the case of *WUS* inactivation, totipotent cells, whose fate is regulated by signals deriving from the organizing center (OC) of the SAM, underwent differentiation in both *Arabidopsis* and *Antirrhinum majus* [37,38]. In *Arabidopsis*, maize, and rice, *WUS* also controls the development of ovules and anthers [39,40].

In the SAM, the activity of *WUS* is controlled by a negative feedback mechanism between the products of the *WUS* and *CLAVATA* (*CLV*) genes [16]. In particular, the CLV3 peptide acts as a mobile signal and, after binding to the CLV1 receptor, can repress *WUS* transcription. An additional negative feedback mechanism, implying both *WUS* and *AGAMOUS* (*AG*) genes, is responsible for the maintenance of floral stem cells. In particular, *WUS* activates the transcription of the *AG* gene, and later, *AG* represses the transcription of *WUS* with a negative feedback mechanism [41].

A *WUS* gene in *Helianthus annuus* (*HaWUS*) was previously characterized [42]. In particular, the lysine 4 (K4) methylation status and acetylation of histone H3 depend on *HaWUS* expression during the zygotic embryo development. The HaWUS recombinant protein [42] bound two copies of the WUS binding site (WUSATA), previously identified on the *LEAFY COTYLEDON1-like* (*HaL1L*) gene [43]. The interaction *HaWUS*/*HaL1L* showed opposite transcriptional behavior during zygotic embryo development. The consequent decrease/increase in positive histone marks bond to both genes suggested an inhibitory effect of *WUS* on *HaL1L* in sunflower zygotic embryos [42]. Until now, *HaWUS* (here renamed *HaWOX1*) is the only member of the WOX family characterized in sunflower.

Sunflower is one of the important oilseed crops grown worldwide as a source of premium oil and dietary fiber that significantly contributes to human health. Many growth and development processes affect sunflower yield. Although *WOX* members have been shown to regulate many aspects of development in several plant species [37,38,39,40], only *HaWOX1* was reported to regulate zygotic embryo development [42,43].

This work aimed to conduct a genome-wide analysis to identify and characterize the entire *WOX* family in this species. The identification and analysis of *WOX* genes in sunflower are supported by completing a complete, reliable genome sequence of *Helianthus annuus*, obtained by third-generation sequencing technology [44]. This study includes the identification of gene families, phylogenetic tree analysis, and the analyses of segmental duplication, gene structure, chromosome location, and expression pattern for members of the *WOX* multigene family in sunflower. Our data will provide a molecular characterization and bioinformatics analysis of *WOX* genes potentially involved in the sunflower developmental processes, including embryonic patterning, ovule development, and inflorescence initiation. The presented results provide a basis for further research on the functional identification and analysis of *WOX* genes in sunflower.

## 2. Results

### 2.1. Identification of HaWOX in Sunflower Genome

Similarity analysis performed by BlastP using as query the known HaWUS (HaWOX1) of sunflower [42] returned 18 candidates WOX protein sequences in sunflower proteome, hereafter reported as HaWOX. The length of HaWOX sequences ranged from 182 to 389 amino acids, composed of a maximum number of five exons (Table S3). Amongst the WOX proteins, 14 encoded for a single gene product whereas two loci (LOC110915545 and LOC110911924) potentially encoded two possible isoforms. A complete list of HaWOX TF sequences is shown in Table S3.

### 2.2. HaWOX Phylogenetic Analysis and Clade Subdivision

**Figure 1:**
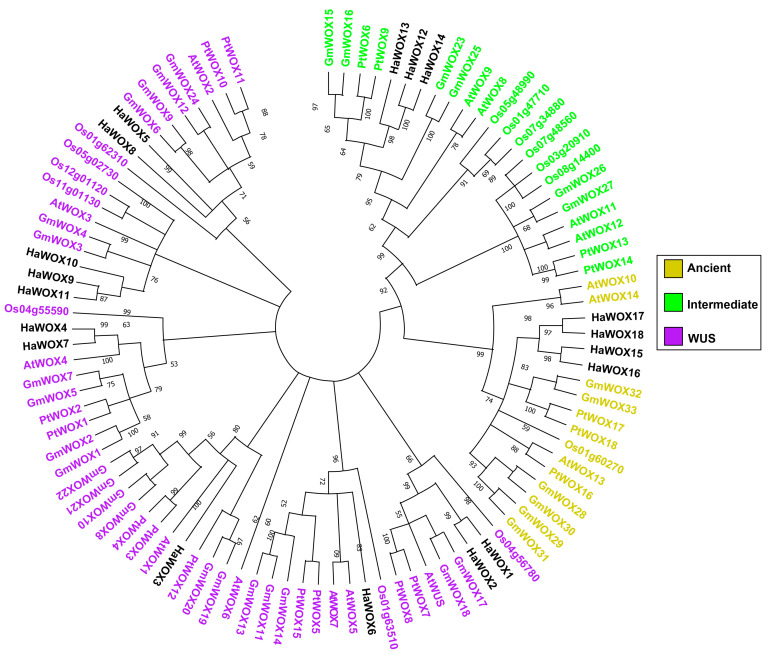
*WOX* phylogenetic tree for *Glycine max* (GmWOX), *Populus trichocarpa* (PtWOX), *Arabidopsis thaliana* (AtWOX), *Oryza sativa* (OsWOX), and *Helianthus annuus* (HaWOX). In the tree, sunflower WOXs are in black characters.

Phylogenetic analysis on protein sequences of HaWOX was performed by using MEGA X. To subdivide into the corresponding ancient, intermediate, and WUS clades, we clustered HaWOX sequences along with a known and characterized WOX protein belonging to *Arabidopsis thaliana*, *Oryza sativa*, *Populus trichocarpa*, and *Glycine max* (Table S1) [45]. Through this analysis, we identified four sunflower WOX sequences belonging to the ancient clade (HaWOX15, HaWOX16, HaWOX17, and HaWOX18), three of the intermediate clade (HaWOX12, HaWOX13, and HaWOX14) and 11 proteins of WUS clade (HaWOX1, HaWOX2, HaWOX3, HaWOX4, HaWOX5, HaWOX6, HaWOX7, HaWOX8, HaWOX9, HaWOX10, and HaWOX11), as shown in Figure 1.

### 2.3. HaWOX Distribution in Sunflower Genome

**Figure 2:**
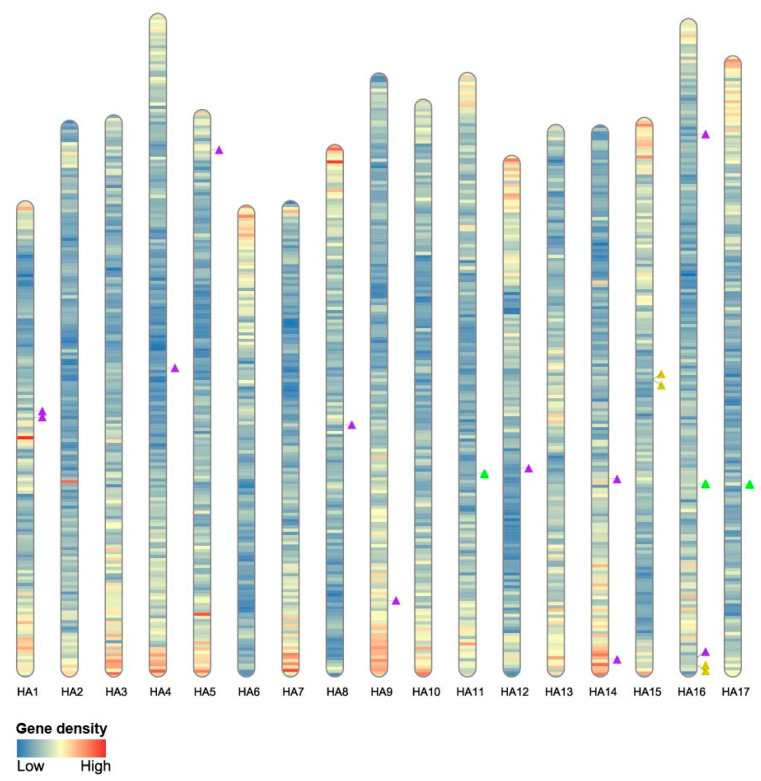
Localization of *HaWOX* genes on the 17 chromosomes of *Helianthus annuus*. Genes corresponding to *HaWOX* sequences are visualized as a triangle for ancient (yellow), intermediate (green), and WUS (purple) clades. HA1-17 = *Helianthus annuus* chromosomes.

The 18 *HaWOX* genes were localized on the 17 chromosomes of haploid genomes of *H. annuus* (Figure 2). Overall *HaWOX* genes were evenly distributed in the sunflower genome; in particular, chromosome 16 showed a major number of sequences with five *WOX* genes (*HaWOX2*, *HaWOX4*, *HaWOX12*, *HaWOX15*, and *HaWOX16*). In contrast, no gene sequences were retrieved in chromosomes 2, 3, 6, 7, 10, or 13 (Figure 2).

Finally, observing gene density divided in the range of 1 Mb in the sunflower genome, *HaWOX* genes were mainly distributed in a low-density area, except for a few members, as in the case of chromosome 14 (Figure 2).

### 2.4. Biochemical, Structural, and Genomic Characterization of Sunflower WOX

Overall protein molecular weight spanned from 21 KDa (HaWOX9) to 43 KDa (HaWOX14) with an average value about 30 KDa.

**Figure 3:**
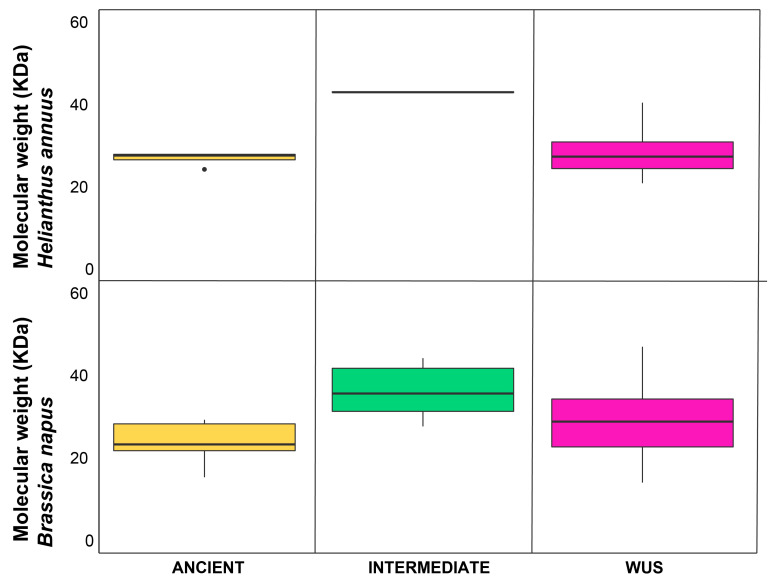
Boxplot of molecular weight for ancient (yellow), intermediate (green), and WUS (purple) HaWOX proteins compared to the corresponding clade of *Brassica napus* [28]. The black line represents the median value per each clade. Black dots represents outlier values.

Regarding clades, HaWOX proteins belonging to the intermediate group showed the highest molecular mass (43 Kodak average), followed by WUS (28 Kodak average) and ancient (26 Kodak average) (Figure 3). Molecular weights of HaWOX proteins were also compared to available data of corresponding clades for *Brassica napus* [28] (Figure 3).

Possible functional domains belonging to HaWOX proteins were investigated by using InterProScan. In particular, the PFAM “Homeodomain” (PF00046) was identified for all the 18 HaWOX sequences; conversely, Panther database distinguished different domains for each clade, such as “PTHR46777:SF4” for intermediate and “PTHR47288:SF1” for ancient group (Table S4). Interestingly, clade WUS showed the greatest variability compared to the other clades, with six different domains identified by Panther in this class (Table S4).

**Figure 4:**
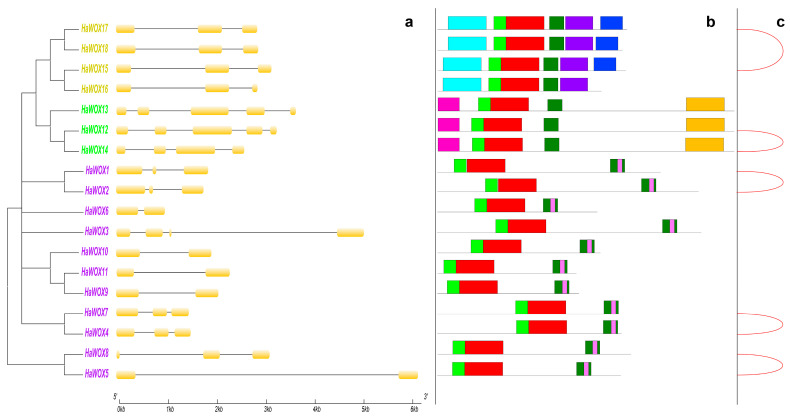
Phylogenetic, genomic, and structural characterization of sunflower *WOX* (*HaWOX*) family subdivided per ancient (yellow), intermediate (green), and WUS (purple) clades. (**a**) = exon (yellow) and intron (black dash) structure for *HaWOX* genes as visualized by GSDS; (**b**) = structural motifs identified on protein sequences by MEME and Clustal Omega (Table S5; Figure 5 and Figure S1); (**c**) = whole genome duplication (red arches) for *HaWOX* multigene family; the edge of the arch indicates the two genes deriving from the same duplication event. Colors in (**b**) are specified in Table S5.

Exon–intron organization for *HaWOX* genes was visualized by using GSDS in order to gain additional information (Figure 4A). Overall, the number of exons ranged from three to five, and a specific pattern was observed for each clade. In particular, the ancient group showed a gene structure formed by three exons and two introns. The intermediate clade’s genes were composed mainly of five exons and four introns, except for the *HaWOX14*. WUS class was the most variable with a global number of exons, which spanned from two to four (Figure 4A).

In *Arabidopsis*, three functional domains (i.e., the WUS-box, the EAR-like motif, and the acidic region) were retrieved in members of the WUS clade. These domains contribute significantly to protein functions [31,46]. In both sunflower and *Arabidopsis* [46,47,48], the WUS-box (TLXLFP), corresponding to a reduced region of the dark-green motif, identified by MEME (Figure 4), was detected in all the WUS-clade members (Figure S1A). In both sunflower and *Arabidopsis* [46], the EAR-like motif (LXLXL) was specific for putative *WUS* genes (Figure S1B). Notably, in sunflower, two TFs, i.e., HaWOX1 and HaWOX2, with the LELXL motif were identified (Figure S1B). According to Salvini et al. [42], CLUSTAL OMEGA alignment identified three putative acidic regions in members of the WUS clade (i.e., HaWOX1, HaWOX2, and HaWOX3) (Figure S2).

**Figure 5:**
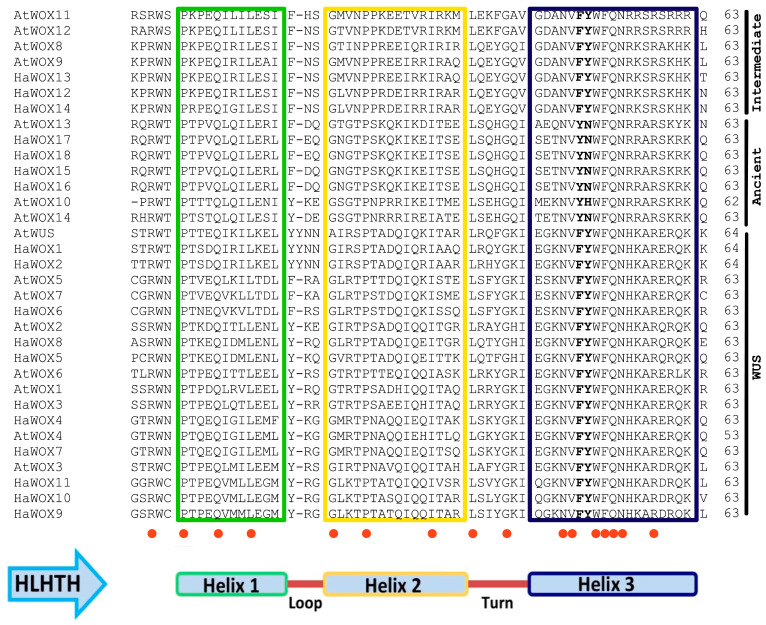
Sequence analysis of homeodomain (HB) in WOX proteins from *H. annuus* (Ha) and *A. thaliana* (At). WOX family members showed the typical helix–loop–helix–turn–helix (HLHTH) structure. Red dots evidence completely conserved residues. The amino-acidic di-residues that discriminate the **FY**WFQ**NH**, **FY**WFQ**NR**, and **YN**WFQ**NR** motifs are indicated in bold characters. These motifs are representative markers for the three main clades, WUS, intermediate, and ancient, respectively.

New structural motifs within HaWOX proteins were explored using MEME. Globally, eight conserved motifs were retrieved, which reflect specific patterns for ancient, intermediate, and WUS clades. A list of conserved motifs is supplied in Table S5. In particular, light-green and red motifs were found in all the 18 analyzed WOX proteins; ancient clade was characterized by two specific motifs (light-blue and purple), similar to the intermediate group (fuchsia and yellow); the WUS clade showed a characteristic motif (dark-green), which marked this class, in addition to a WUS-box (pink motif) retrieved exclusively in WUS clade (Figure 4B). Our results showed that the red motif (Figure 4B) represents a part of the highest conserved region of the homeodomain (HB) that contains the typical helix–loop–helix–turn–helix (HLHTH) structure, which was either 63 or 64 amino acid residues in length (Figure 5). In sunflower, the **FY**WFQ**NH**, **FY**WFQ**NR**, and **YN**WFQ**NR** motifs (from the 50th to 56th amino acid in the HB domain) identified the three main clades: WUS, intermediate, and ancient, respectively. We did not observe intron insertions in the HB domains of the 18 *WOX*s from sunflower (Figure S3).

### 2.5. HaWOX Genes Duplication Events in Sunflower Genome

**Figure 6:**
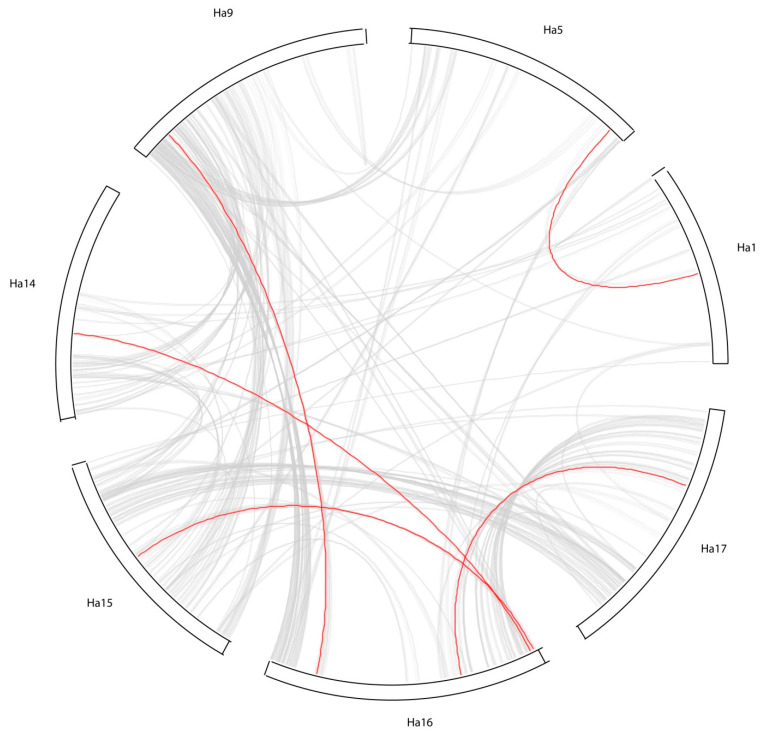
Graphical representation of synteny blocks of *Helianthus annuus* chromosomes containing *HaWOX* genes originated in whole genome duplication events. Red arches join a two-synteny block containing *WOX* originated by whole genome duplication events. Grey arches show synteny blocks for analyzed chromosomes.

MCScanX was used to investigate possible duplication events for the *WOX* genes to explore the evolution of this multigene family in the sunflower genome. Analysis evidenced events of whole genome duplication (WGD) for five pairs of sunflower *WOX* family, in particular: *HaWOX1–HaWOX2*, *HaWOX4–HaWOX7*, *HaWOX5–HaWOX8*, *HaWOX12–HaWOX14*, and *HaWOX17–HaWOX15* (Figure 6).

Interestingly, MCScanX marked the other *HaWOXs* as duplicated and dispersed elements in the sunflower genome. These data indicate that segmental duplication has contributed to the diversity and expansion of *HaWOX* gene families.

The nonsynonymous/synonymous ratio (Kn/Ks) was used to detect evolutionary constraints amongst *HaWOX* gene pairwise duplication that mostly showed a strong purifying selection (Table S6).

### 2.6. HaWOX Gene Expression

Quantitative RT examined the expression patterns of 16 *HaWOX* genes to investigate the details of organ-specific expression of the *HaWOX* genes in sunflower.

**Figure 7:**
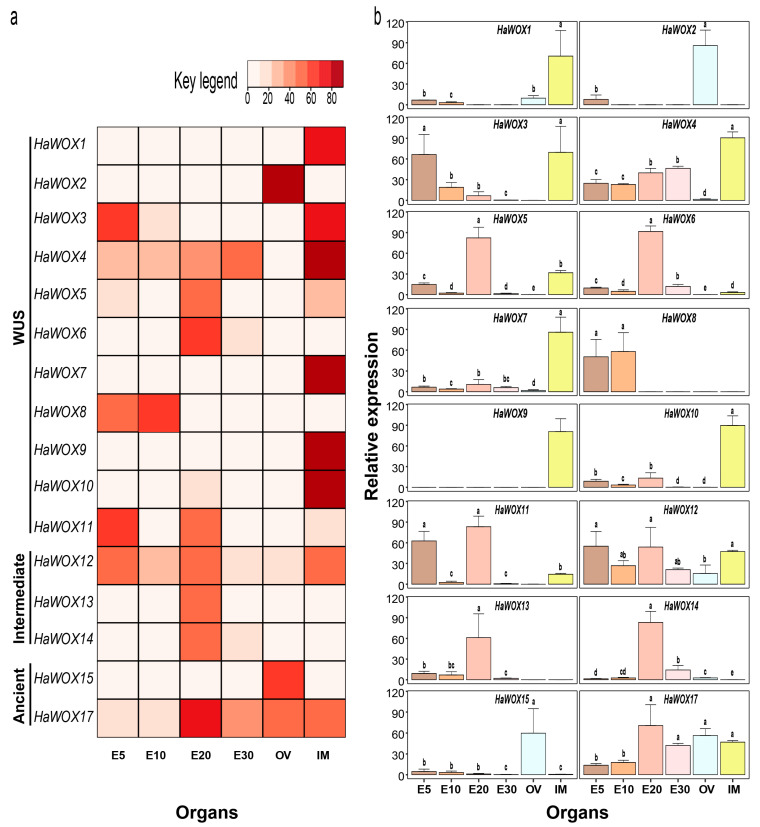
Expression profile of *H. annuus WUSCHEL HOMEOBOX-like* (*HaWOX*) in the ovule (OV), inflorescence meristem (IM), and at different stages of embryo development (E5, E10, E20, and E30). (**a**) Heatmap graph of *HaWOX* relative expression, scale range from dark to light red for higher and lower expressed genes, respectively. (**b**) Bar plots of *HaWOX* relative expression: heights for each gene indicate relative differences in expression level; bars show the average value for three biological replicates. Different letters indicate statistically significant differences between organs after one-way ANOVA according to Tukey’s HSD post hoc test (*p* < 0.05).

PCR (qRT–PCR) in three organs, i.e., embryos at 5, 10, 20, and 30 DAP (E5, E10, E20, and E30), ovules one day before pollination (OV) and inflorescence meristems (IMs) from 35–40-day-old plants (Figure 7a,b). Although we used several combinations of primers, we were unable to generate correct (multiple peaks in the melt curve) *HaWOX16* and *HaWOX18* amplicons.

The results showed that several WUS clade members (i.e., *HaWOX1*, *HaWOX7*, *HaWOX9*, and *HaWOX10*) were mainly expressed in inflorescence meristems. Moreover, transcripts of *HaWOX3* (WUS clade) were abundant in inflorescence meristems; in addition, *HaWOX3* expression was detected during zygotic embryo development, especially in the early stages (E5). *HaWOX4*, a WUS clade member, was highly accumulated in inflorescence meristems and in all embryo developmental stages, where its expression reached the highest level at advanced embryo maturation (E30). Transcripts of *HaWOX5* and *HaWOX6* genes from the WUS clade, and transcripts of *HaWOX13* and *HaWOX14* from the intermediate clade, mainly accumulated in the E20 embryo developmental stage. The WUS clade member *HaWOX8* was specifically expressed in the first stages of embryo development (i.e., E5 and E10). In contrast, transcripts of *HaWOX11*, another WUS clade member, were highly accumulated in E5 and E20 embryo development stages. Interestingly, a member of the intermediate clade (*HaWOX12*) and a member of the ancient clade (*HaWOX17*) showed a relatively high transcript accumulation in all analyzed organs (Figure 7). The ancient clade member *HaWOX15* was mainly expressed in the mature ovule, similar to the *HaWOX2* gene, a WUS clade member.

## 3. Discussion

### 3.1. Structural Conservation of the Sunflower WOX Gene Family

*WOX* genes encode for TFs, which belong to the homeobox superclass; these genes play important roles in plant development and the response to various biotic and abiotic stresses [22,23,28], as well as in plant growth regulators [29]. Therefore, this class of TFs has been identified and explored in several species, including *Oryza sativa*, *Sorghum bicolor*, *Arabidopsis thaliana*, *Populus trichocarpa*, *Zea mays* [46], *Brassica napus* [28,49], *Jatropha curcas* [50], cotton (*Gossypium arboreum, G*. *raimondii*, and *G. hirsutum*) [51], *Solanaceae* [52], *Camelia sinensis* [53], *Glycine max* [54], *Pinus pinaster* [55], *Vitis vinifera* [56], *Picea abies* [57], *Salix suchowensis* [58] *Pyrus bretschneideri*, *Prunus persica*, *Prunus mume*, and *Fragaria vesca* [59]. However, there are still no genome-wide analyses concerning the *WOX* genes in the Asteraceae family, the most prominent family belonging to the angiosperms, and specifically in a crop with a considerable economic interest, such as the sunflower.

The sunflower genome sequence provided valuable information for the structural and functional analysis of *H. annuus* genes [44]. This information is crucial for a reliable analysis of multigenic families, specifically focusing on *HaWOX* genes. In total, 18 *HaWOX* members were identified in the sunflower genome. The phylogenetic analyses, performed by comparing the sunflower sequences with well-known sequences of WOX proteins characterized in other species, have shown that the distribution in the three clades (ancient, intermediate, and WUS), distinctive of the multigene *WOX* family [26,46,47], is also conserved in sunflower.

The monophyletic origin of the multigene *WOX* family showed that the last common ancestor to green algae and terrestrial plants features at least one *WOX* gene [60]. The ancient clade is present in all the main plant lineages, including green algae and lower plants [60]. These observations indicate that ancient is the most ancestral and preserved clade [26,61]. In particular, WOX proteins belonging to the ancient clade were also identified in *H. annuus* (i.e., HaWOX15, HaWOX16, HaWOX17, and HaWOX18). Notably, these proteins are gene isoforms.

The *WOX* gene family has undergone a great expansion after the separation of bryophytes from other terrestrial plants. In fact, the number of *WOX* genes increased with the development of vascular plants [25,61]. Changes in the three-dimensional structure of the HD and the appearance of specific motifs in the protein sequence may also have contributed to the functional changes of the *WOX* family during evolution [60].

The Intermediate clade, found in all vascular plants and WUS clade, retrieved exclusively in ferns, gymnosperms, and angiosperms, originated from the members of the ancient clade after gene duplication and subsequent modifications [60]. In particular, the WUS clade has expanded and evolved in spermatophytes, leading to many members with consequent functional and structural diversification [60].

In sunflower, there are three *HaWOX* genes belonging to the intermediate clade: *HaWOX12*, *HaWOX13*, and *HaWOX14*. Instead, the WUS clade shows a higher number of members than other clades, with a total of 11 genes, characterized by greater structural variability compared to the intermediate and ancient clade. This finding agrees with data reported for the WUS clade in other dicotyledonous species [46,49,62].

Sunflower WOX clades showed specific structural motifs capable of distinguishing each group. These results agree with the studies by Lian et al. [60] on the origin and evolution of the WOX protein family in the plant kingdom. Notably, we detected that the functional conservation of each clade was also supported by a highly conserved gene structure, as shown by exon–intron patterns exhibited by the three clades. The HB domain contains a helix–loop–helix–turn–helix structure [27], which can distinguish sequence-specific targets in a precise spatial and temporal organization. Furthermore, the HB domain is conserved in different species, thus maintaining its functional integrity [37,47]. Indeed, the **FY**WFQ**NH**, **FY**WFQ**NR**, and **YN**WFQ**NR** motifs (from 50th to 56th amino acid in the HB domain) have been reported as representative markers for the three main clades WUS, intermediate, and ancient, respectively [28]. The presence of these motifs was also retrieved in sunflower WOX sequences. Altogether, our protein sequence analysis indicated that the HB domains are highly conserved in HaWOX proteins. However, according to Wang et al. [28], we did not observe intron insertions in the HB domains. In addition, WUS members of the *HaWOX* family also contain a canonical WUS-box motif within the core sequence TL–LFP. Accordingly, in *Arabidopsis*, the WUS-box existed in most members of the WUS clade, except those in the WOX7 subclade [26]. It was shown that the WUS-box interacts with TOPLESS-type corepressors (*AtWUS*, *AtWOX1*, and *AtWOX5*) [37,63,64] to mediate gene repression via histone deacetylation (*AtWOX5*) [64]. These results agree with genome-wide analyses of the *WOX* multigene family in several plant species [28,29,59,65]. Furthermore, the EAR motif was identified in two HaWOX TFs belonging to the WUS clade (i.e., HaWOX1 and HaWOX2); notably, these two WOX proteins showed the highest homology within the WUS clade, and whose 64 amino acid residues constitute the HB homeodomain similar to AtWUS. In *Arabidopsis*, *AtWUS* is considered the prototypic of the *WOX* family [24]; therefore, we can hypothesize that *HaWOX1* and *HaWOX2* could be prototypic of the *WOX* family in sunflower. Moreover, although we do not have data on the *HaWOX2* gene, in situ, hybridization unequivocally demonstrates the localization of *HaWOX1* in the few cells of the OC of sunflower SAM [42]. Similarly, *AtWUS* RNA is found in a few cells of the OC located just beneath the central zone of the SAM [24]. Restriction of *AtWUS* transcription to cells of the OC is critical for maintaining a constant number of stem cells [16]. In *Arabidopsis*, the EAR motif has been found in others WUS clade members (AtWOX5 and AtWOX7), also involved in transcriptional repression [66].

Finally, the acidic region of AtWUS, localized at the N-terminal side of the WUS-box, was not strictly conserved in sunflower WUS clade sequences, as shown by our analysis. Indeed, few HaWOX proteins (i.e., HaWOX1, HaWOX2, and HaWOX3) showed putative acidic motifs. These results are consistent with previous findings, suggesting that the acidic region domain could play an important role mainly in *Arabidopsis* WOX TFs [46]. In particular, the acidic domain, identified in AtWUS clade members (i.e., AtWUS, AtWOX4, AtWOX5, AtWOX6, and AtWOX7), has been proposed as a potential transcriptional activation domain for eukaryotes [67].

Molecular characterization also showed that the HaWOX proteins of each clade could be distinguished by molecular weight, which is similar within the different groups; this is particularly evident in intermediate and ancient clades, while the WUS clade is characterized by greater variability as already detected in *Brassica napus* [28].

The structure of the sunflower genome is particularly complex due to the evolutionary history of asterids, in which a whole-genome triplication was characterized at the base of the asterid II clade and a sunflower-specific WGD around 29 million years ago [44]. Duplication events that bring sunflower genome evolution are consistent with our results, which underlined an extensive WGD occurring amongst *HaWOX* genes. *HaWOX* genes are homogeneously distributed on the 17 chromosomes of *H. annuus*, and in particular, five *HaWOX* gene pairs resulting from WGD-like events were found, showing similar characteristics. The pairs detected are composed of *HaWOX* genes of the same clade: *HaWOX1–2*, *HaWOX4–7*, and *HaWOX5–8* for the WUS clade; *HaWOX12–14* for the intermediate clade; *HaWOX15–17* for the ancient clade. *HaWOX1–2* and *HaWOX4–7* have an identical organization in introns (two) and exons (three); moreover, the same signature “PTHR45940: SF2” identifies *HaWOX1–2* while the signature “PTHR47716” identifies *HaWOX4–7*. In the phylogenetic tree, *HaWOX5–8* form a distinct cluster compared to the other *HaWOX* members. They are characterized by three and two exons, respectively, and share the same signature, “PTHR45940: SF6”. *HaWOX12* and *HaWOX14* contain five and four exons, respectively, and share the same signature, “PTHR47288: SF1”. Furthermore, they present all the motifs characteristic of the intermediate clade. *HaWOX15–17* contains three exons sharing the same signature, “PTHR46777: SF4”, also featuring motifs characteristic of the ancient clade. The occurrence of genes originating from WGD duplication within the *WOX* family was also detected in other plant species, such as *Gossypium* [51] and Rosaceae species [58], whose genome was subject to large-scale or even genome-wide duplication events that occurred in their ancestors.

The nonsynonymous/synonymous ratio between duplicated *HaWOX* genes indicates no positive selective pushes at the origin of the *HaWOX* genes. Therefore, the strong purifying selection evidenced by results suggests that mutations in these genes could be disadvantageous if fixed in the sunflower genome; this may mean that the structure and sequences of *HaWOX* genes are preserved in the genome, given the crucial functions they perform from the early stages of both embryo and flower development. The effects of mutations affecting members of this multigene family have been analyzed in other species, showing that loss of function profoundly compromised the development of SAM and RAM [24,35] and adversely affected flower [24] and fruit development [68].

Despite their function as transcription factors, no clear nuclear localization signal (NLS) can be predicted for any of the *WOX* family members [26]. In *Arabidopsis*, *WUS WOX6* and *WOX11* are localized in the nucleus [26]. Nuclear localization has also been demonstrated for other *WOX* genes in other species (e.g., *Jatropha curcas*, *Nicotiana tabacum, Broussonetia kazinoki* × *Broussonetia papyrifera*) [46,52,69]. The cellular localization of *HaWOX* genes will be the subject of future work.

### 3.2. Gene Expression Analysis

The expression patterns of *HaWOX* genes, obtained using quantitative qRT–PCR, suggested that members of the *HaWOX* family might function in different key aspects of sunflower embryo development, ovule growth, and inflorescence initiation. In situ hybridization on sunflower zygotic sections of embryos at 10 and 26 DAP revealed that *HaWUS* (here renamed *HaWOX1*) marks the OC region in the SAM [42]. This expression pattern was consistent with the results observed in zygotic embryos and postembryonic tissues of *Arabidopsis* [37]. Furthermore, results from RT–qPCR performed during this work showed that both genes appear to have transcriptional activity in the early stages of embryonic development. Notably, a high *HaWOX1* transcript level was detected in IMs of sunflower. Similarly, *AtWUS* is required during the development of female and male organs [39,40], which acts through positive regulation of *WINDHOSE* gene expression [70]. In addition, for determinate floral meristems, *AtWUS* directly activates transcription of the *AG* gene, repressing *AtWUS* [41], leading to the termination of the stem cells at the end of flower development. In sunflower, many other WUS clade members (i.e., *HaWOX3*, *HaWOX4*, *HaWOX5*, *HaWO7*, *HaWOX9*, and *HaWOX10*) were highly expressed in IMs, suggesting a pivotal role for these genes in flower initiation and also in the organization of inflorescence architecture. A high expression of WUS clade genes in flowers was also detected for *Oryza sativa* [60]. Furthermore, the expression of *HaWOX3*, *HaWOX4*, *HaWOX5*, *HaWOX7*, and *HAWOX10* was also retrieved with high expression levels in different stages of embryo development. For example, transcripts of *HaWOX3* were mainly accumulated in the first days after pollination (DAP). By contrast, many *HaWOX* genes showed the highest expression levels in embryos at 20 DAP. Similar evidence (highest expression levels at 20 DAP) was observed for WUS clade members *HaWOX5* and *HaWOX6*, together with members of the intermediate clade (i.e., *HaWOX11*, *HaWOX12*, *HaWOX13*, and *HaWOX14*) and ancient clade (*HaWOX17*). In sunflower, this developmental stage is critical for embryo maturation. In addition, the abscisic acid (ABA) content increased sharply, reaching the highest level at 20 DAP in sunflower embryos [71]; ABA is fundamental to establishing both seed dormancy and the response of plants to dehydration stress [71,72,73]. Furthermore, in embryos, the highest concentration of oil body-per-cell number was detected at 20–25 DAP [74], and the heat-shock TF HaHSFA9 showed the highest accumulation between 18 and 21 DAP; this TF is involved in seed development [62]. Several *HaWOX* genes are likely involved in some of the key events detected in sunflower embryos around 20 DAP. The *HaWOX2* gene (WUS clade) is specifically expressed in the ovule, similar to a *WUS* gene in *Arabidopsis* [75]. Notably, the transcripts of ancient clade member *HaWOX15* are also accumulated in the ovule one day before pollination. The expression of *HaWOX8* was detected in the first stages of embryo development (i.e., 5 and 10 DAP), as observed in *Arabidopsis* [75].

It is worth noting that although many *HaWOX* genes originate by WGD duplication or possible segmental duplication sharing coding sequence and structural features, they showed different expression patterns, indicating that during sunflower evolution, these paralogous genes had enough time to differentiate at the expression level and possibly at a functional level. If genes *HaWOX4* and *HaWOX7* showed a similar expression pattern, other genes such as *HaWOX1* and *HaWOX2*, probably originating from WGD duplication, showed different expression patterns.

## 4. Materials and Methods

### 4.1. WOX Identification in Sunflower Genome

The genome annotation file with transcripts and protein sequences for *H. annuus* v. 2.0 was downloaded from the National Center for Biotechnology Information (NCBI; https://www.ncbi.nlm.nih.gov, accessed on 15 July 2020). WOX sequences were identified in the sunflower proteome by using blastP (BLAST package v2.6.0+) using a known sequence of sunflower HaWUS as a query [42], available at accession number LN811433. In particular, the homology search was set to 40 percent of sequence similarity and the E-value to 10E-3. Sequences identified by blastP were marked as *H. annuus* WOX (HaWOX). Further investigation on HaWOX sequences was performed by using InterProScan [76] using the Pfam23.0 and Panther version 17 (http://pantherdb.org, accessed on 6 December 2022) databases in order to find possible common protein domains for the *WOX* gene family.

### 4.2. Alignments and Phylogenetic Analysis for HaWOX

Protein sequences of candidate HaWOX were aligned with other known WOX proteins of *Arabidopsis thaliana*, *Oryza sativa*, *Populus trichocarpa*, and *Glycine max* reported in a previous study (Table S1) [45]. Alignment was performed using Muscle v. 3.8 on MEGA X v. 10.0 (htpp://www.megasoftware.net, accessed on 6 December 2022) with default parameters [77]. The phylogenetic tree was constructed on MEGA X using the maximum likelihood parameter with total of 1000 bootstrap replications performed by Jones, Taylor, and Thornton (JTT) amino acid substitution model.

### 4.3. Structural and Genomic Characterization of HaWOX Sequences

New possible motif structures in HaWOX protein sequences were investigated by using the web version of multiple expectation maximization for motif elicitation (MEME, v. 5.5.1) [78]. Analysis was performed by using default parameters except for “number of motifs”, which was set to eight. In addition, Clustal Omega v.1.2.4 and ClustalW2 v. 2.1 were used to identify some basic motifs of *WOX* genes unidentified by MEME. Concerning *HaWOX* gene sequences, the Gene Structure Display Server (GSDS v.2.0; http://gsds.gao-lab.org/, accessed on 6 December 2022) was exploited to display the exon–intron structure by supplying to the software gene and CDS sequences in FASTA format combined with Newick file, provided by MEGA X, in order to keep phylogenetic order of *HaWOX*. EMBOSS Pepstats v. EMBOSS:6.4.0.0 [79] with default options was used to calculate molecular weight (Da) from HaWOX amino acid sequences.

### 4.4. HaWOX Chromosomal Distribution and Gene Duplication Events

Possible duplication events for *HaWOX* multigene family were analyzed by using MCScanX v. 1.0 [80]. The file containing annotation and protein sequence similarity was constructed by using BlastP and sunflower annotation file (GFF). Tandem and gene duplication events for *HaWOX* were obtained from the collinearity file by using the perl script named “detect_collinearity_within_gene_families” provided by MCScanX. The “Rideogram” R package was exploited to visualize HaWOX distribution in sunflower chromosomes graphically [81]; gene locations and gene density per each chromosome were obtained using the GFF file provided by Badouin et al. [44] to NCBI. Evolutionary constraints expressed as the ratio between nonsynonymous and synonymous substitution (Kn/Ks) for pairwise comparison of HaWOX protein sequences were detected using PAL2NAL [82]; alignment between protein pairs was generated with Clustal Omega.

### 4.5. Gene Expression Analysis by Quantitative Real-Time Quantitative PCR (qRT–PCR)

Seeds of the inbred line CM of *Helianthus annuus* L. (DAFE, University of Pisa, Italy) were germinated in Petri dishes on paper discs soaked in distilled water at 23 ± 1 °C. Seedlings were transplanted into 20 cm diameter pots containing a mixture of soil and sand. As described previously, plants were grown under standard growth chamber conditions [83]. Total RNA was extracted from 100 mg of fresh inflorescence meristem (IM) of 35–40-day-old plants from ovules (OV) one day before pollination and from embryos at 5, 10, 20, and 30 days after pollination (DAP, E5, E10, E20, and E30). Following the manufacturer’s instructions, the extraction was performed using RNeasy Plant Mini Kit (Qiagen GmbH, Hilden, Germany). According to Fambrini et al. [83], the extracted RNA samples were also treated with Amplification Grade DNAse I (Sigma-Aldrich, St. Louis, MO, USA). The concentration of each RNA sample was measured using Qubit RNA BR Assay Kits (Applied Biosystems Invitrogen, Life Technology Corporation, Eugene, OR, USA). RNA purity was assessed by determining the spectrophotometric absorbance at 230, 260, and 280 nm and the ratios of *A*260:*A*280 and *A*260:*A*230. RNA integrity was evaluated by 28S/18S ribosomal RNA (rRNA) ratio after agarose gel electrophoresis. The RNA samples (400 ng) were reverse-transcribed into cDNA employing the Maxima First Strand cDNA Synthesis Kit (Thermo Fisher Scientific, Vilnius, Lithuania). The synthesized cDNA was used for quantitative real-time quantitative PCR (qRT–PCR) using gene-specific primer pairs (Table S2). Real-time PCR was performed in the presence of Fast SYBRTM Green Master Mix (Applied Biosystems, Thermo Fisher Scientific, Vilnius, Lithuania), with the real-time StepOnePlus™ apparatus (Applied Biosystem, Thermo Fisher Scientific Waltham, MA, USA), according to the recommended thermal cycling conditions. Here, *actin* [54] and *tubulin alpha* (NCBI reference sequence: XM_022136836.2) were selected as housekeeping genes. Although all the endogenous control genes tested exhibited stable expression among the different samples, actin was chosen to normalize gene expression data for its high transcriptional stability [84]. The primers used for qRT–PCR were designed using Primer-BLAST software (https://www.ncbi.nlm.nih.gov/tools/primer-blast/, accessed on 6 December 2022) listed in Table S2. The amplification of the target genes and the endogenous controls were run using three biological replicates, each with three technical replicates and were analyzed on the same plate in separate tubes. The transcripts’ relative abundance was calculated using the 2-DD*C*T method [85]. For the analysis of *HaWOX* gene expression levels in sunflower organs, the highest expression value encountered in the tested samples was set to 100 for each gene separately, and lower values were normalized to this value according to Tähtiharju et al. [84]. Before the quantification, a preliminary experiment was performed to ensure that the amplification efficiencies of the target and the reference genes were similar. qRT–PCR data were graphically visualized by heatmap, generated using the function “heatmap.2” available in the R v. 4.1.0 package “gplots” v. 3.1.3 (http://cran.ma.imperial.ac.uk/web/packages/gdata/, accessed on 6 December 2022).

### 4.6. Statistical Analysis

In the expression analysis, the values are means (±SD) from three different RNA replicates for each organ type. Data from morphological and qRT–PCR analyses were treated using the ANOVA test (analysis of variance between groups). All the means were separated using Tukey’s HSD (honestly significant difference) post hoc test (*p* ≤ 0.05). The normality of data was tested using the Shapiro–Wilk test, whilst the homoscedasticity was tested using Bartlett’s test. This statistical analysis was conducted using GraphPad (GraphPad, La Jolla, CA, USA).

## 5. Conclusions

In conclusion, these results suggest that *HaWOX* family members, diversified during the evolution of sunflower, might be involved in many aspects of sunflower embryo growth and development as well as ovule and inflorescence meristem differentiation and organization. Several *WOX* genes (i.e., *HaWOX4*, *HaWOX5*, HaWOX6, *HaWOX11*, *HaWOX12*, *HaWOX13*, *HaWOX14*, and *HaWOX17*) are highly expressed in 20-day embryos; this is a critical period for the accumulation of ABA and water stress-related proteins [81,82,83,85]. Their functional study should provide critical information for the genetic improvement of drought resistance in sunflower genotypes. Notably, this is the first work to study this TF family, which plays key roles in sunflower development at the genome-wide level. Furthermore, as for the *HaWOX1* gene [42,43], other investigations (e.g., in situ hybridization, the analysis of promoters, methylation levels, interactions with other genes, and the analysis of additional organs) are essential to understanding the specific role of each *HaWOX* gene.

## Data Availability

Data are contained within the article or in supplementary materials.

## Supplementary Materials

The following supporting information can be downloaded at: https://www.mdpi.com/article/10.3390/ijms24043352/s1.

## Abbreviations

ABA	Abscisic acid
*AG*	*AGAMOUS*
*CLV*	*CLAVATA*
DAP	Days after pollination
E	Embryo
EAR	ERF-associated amphiphilic repression
IM	Inflorescence meristem
*HaL1L*	*LEAFY COTYLEDON1-Like*
HB	*WOX homeodomain*
HLHTH	Helix–loop–helix–turn–helix
*Kn-1*	*Knotted-1*
OC	Organizing center
O	Ovule
qRT–PCR	Quantitative real-time polymerase chain reaction
RAM	Root apical meristem
SAM	Shoot apical meristem
TF	Transcription factor
*TPL*	*TOPLESS*
*WOX*	*WUSCHEL-related homeobox genes*
*WUS*	*WUSCHEL*
