# Supplementary material for: Genome-Wide Analysis of WOX Multigene Family in Sunflower (Helianthus annuus L.)
Source: Int J Mol Sci. 2023 Feb 8;24(4):3352. doi: 10.3390/ijms24043352 (PMC9968055; doi:10.3390/ijms24043352)
Supplement: Supplementary file 1 [file ijms-24-03352-s001.zip › ijms-2115791-supplementary.pdf]

Supplementary Materials

Article

Genome-Wide Analysis of *WOX* Multigene Family in Sunflower (*Helianthus annuus* L.)

Ettore Riccucci [1,2,†], Cosimo Vanni [1], Alberto Vangelisti [1,†], Marco Fambrini [1], Tommaso Giordani [1], Andrea Cavallini [1], Flavia Mascagni [1,*] and Claudio Pugliesi [1]

[1] Department of Agriculture Food and Environment, University of Pisa, Via del Borghetto 80, 56124 Pisa, Italy
[2] Scuola Superiore Sant'Anna, Piazza Martiri della Libertà 33, 56127 Pisa, Italy
* Correspondence: flavia.mascagni@unipi.it
† These authors contributed equally to this work.

Glycine max			*Arabidopsis thaliana*			*Oryza sativa*			*Populus trichocarpa*		
1	GmWOX01	Glyma01g37190.2	1	AtWOX14	AT1G20700.1	1	OsWOX01	LOC_Os01g47710.1	1	PtWOX1	Potri.014G025300.1.p
2	GmWOX02	Glyma02g10410.1	2	AtWOX10	AT1G20710.1	2	OsWOX02	LOC_Os01g60270.1	2	PtWOX2	Potri.002G124100.1.p
3	GmWOX03	Glyma02g42200.1	3	AtWOX04	AT1G46480.1	3	OsWOX03	LOC_Os01g62310.1	3	PtWOX3	Potri.012G047700.1.p
4	GmWOX04	Glyma03g01000.1	4	AtWOX06	AT2G01500.1	4	OsWOX04	LOC_Os01g63510.1	4	PtWOX4	Potri.015G039100.1.p
5	GmWOX05	Glyma04g01830.1	5	AtWUS	AT2G17950.1	5	OsWOX05	LOC_Os03g20910.1	5	PtWOX5	Potri.010G192100.1.p
6	GmWOX06	Glyma04g04310.1	6	AtWOX03	AT2G28610.1	6	OsWOX06	LOC_Os04g55590.1	6	PtWOX6	Potri.011G061400.1.p
7	GmWOX07	Glyma05g33850.1	7	AtWOX09	AT2G33880.1	7	OsWOX07	LOC_Os04g56780.1	7	PtWOX7	Potri.005G114700.1.p
8	GmWOX08	Glyma06g01940.1	8	AtWOX11	AT3G03660.1	8	OsWOX08	LOC_Os05g02730.1	8	PtWOX8	Potri.007G012100.1.p
9	GmWOX09	Glyma06g04470.1	9	AtWOX05	AT3G11260.1	9	OsWOX09	LOC_Os05g48990.1	9	PtWOX9	Potri.004G051600.4.p
10	GmWOX10	Glyma07g11372.1	10	AtWOX01	AT3G18010.1	10	OsWOX10	LOC_Os07g34880.1	10	PtWOX10	Potri.009G029200.1.p
11	GmWOX11	Glyma07g15710.2	11	AtWOX13	AT4G35550.1	11	OsWOX11	LOC_Os07g48560.1	11	PtWOX11	Potri.001G237900.1.p
12	GmWOX12	Glyma07g32430.1	12	AtWOX07	AT5G05770.1	12	OsWOX12	LOC_Os08g14400.1	12	PtWOX12	Potri.010G111400.1.p
13	GmWOX13	Glyma07g34425.1	13	AtWOX12	AT5G17810.1	13	OsWOX13	LOC_Os11g01130.1	13	PtWOX13	Potri.013G066900.2.p
14	GmWOX14	Glyma08g05831.1	14	AtWOX08	AT5G45980.1	14	OsWOX14	LOC_Os12g01120.1	14	PtWOX14	Potri.019G040800.2.p
15	GmWOX15	Glyma09g30831.1	15	AtWOX15	AT5G46010.1				15	PtWOX15	Potri.008G065400.1.p
16	GmWOX16	Glyma10g08030.1	16	AtWOX02	AT5G59340.1				16	PtWOX16	Potri.005G101800.1.p
17	GmWOX17	Glyma10g43580.2							17	PtWOX17	Potri.005G252800.1.p
18	GmWOX18	Glyma11g08091.1							18	PtWOX18	Potri.002G008800.1.p
19	GmWOX19	Glyma11g14940.2									
20	GmWOX20	Glyma11g34990.1									
21	GmWOX21	Glyma12g06895.1									
22	GmWOX22	Glyma13g21860.2									
23	GmWOX23	Glyma13g24150.1									
24	GmWOX24	Glyma13g41000.1									
25	GmWOX25	Glyma14g09310.1									
26	GmWOX26	Glyma15g04460.1									
27	GmWOX27	Glyma17g35880.2									
28	GmWOX28	Glyma18g03350.1									
29	GmWOX29	Glyma18g39520.2									
30	GmWOX30	Glyma18g52491.1									
31	GmWOX31	Glyma19g29660.2									
32	GmWOX32	Glyma20g02161.1									

33	GmWOX33	Glyma20g23220.2									

Table S1. Accession numbers of *WOX* genes from different species (i.e., *Glycine max*, *Arabidopsis thaliana*, *Oryza sativa*, and *Populus trichocarpa*) whose amino acid sequences were used for the Neighbor-Joining phylogenetic tree.

Gene Name	NCBI gene ID	NCBI protein ID	NCBI mRNA ID	Primer name	Primer sequence (5'-3')	bp	Tm	Amp
Actin-7 (chr 15)	LOC110909803	XP_022010246.1	XM_022154554.2	AF	GTATTGTCAGCAACTGGGATGA	22	58,4	108
				AR	ATGGTGCCTCAGTGAGAAGAAC	22	60,3	
Alpha-Tubulin (chr 5)	LOC110941095	XP_022038405.2	XM_022182713.2	TUBF	TCTTCTGTTGGAGCGTCTGTCG	22	62,1	124
				TUBR	GTGGGTGGAAAGCACACTGTTG	22	62,1	
HaWOX1 (chr 14)	LOC110905747	XP_022006965.1	XM_022151273.2	F1_1	TCATCACATTGGGGTTGGTTC	21	57,9	115
				R1_1	CTCTCCCGGTGGTGATATTGA	20	57,3	
HaWOX2 (chr 16)	LOC110918187	XP_022018228.1	XM_022162536.2	F2_2	GGTGGTTCTTCATCTTCACAAG	22	58,4	109
				R2_2	CTGTGTACTCTCCCGGTGAT	20	59,3	
HaWOX3 (chr 12)	LOC110895832	XP_021998889.1	XM_022143197.2	F1_3	TGACGTGGACTCTTATGAAACCC	23	60,6	139
				R1_3	GGGTAGAAACTCGATGAACTGTC	23	60,6	
HaWOX4 (chr 16)	LOC110917230	XP_022017513.1	XM_022161821.2	F1_4	CCCATCCTCCACCATGATAAACT	23	60,6	97
				R1_4	GCACTCAAATGACCAACTTCTAC	23	58,9	
HaWOX5 (chr 1)	LOC110943319	XP_022040761.1	XM_022185069.2	F2_5	CCACAACGTGACATAGGGTTCTA	23	59,8	158
				R2_5	CTTGGTTCGGGTGGTGGTC	19	58,4	
HaWOX6 (chr 14)	LOC110906266	XP_022007144.1	XM_022151452.2	F1_6	CAAACCTAGCCGCAGTACTTCTA	23	60,6	169
				R1_6	CTTACCATAAAAGCTCAACTGGG	23	58,9	
HaWOX7 (chr 9)	LOC110879415	XP_021983563.1	XM_022127871.2	F5_7	TGGAGATGTTATATAGAGGTGGA	23	57,1	157
				R5_7	TTGCCATACTTGCTTAGTTGAGA	23	57,1	
HaWOX8 (chr 5)	LOC110942716	XP_022040174.1	XM_022184482.1	F1_8	GCCGTTTGTTCATATACCTCC	21	57,9	130
				R1_8	GTCGGTTGAGAGAGCTGCT	18	58,8	
HaWOX9 (chr 4)	LOC110934532	XP_022033204.1	XM_022177512.2	F1_9	CCTCTTCATCAGGTTGCTGG	20	59,3	103
				R1_9	ATGTGAGTTTGCAGGTGCTATC	22	58,4	
HaWOX10 (chr 8)	LOC110871325	XP_021975839.1	XM_022120147.2	F2_10	CAACTCACCTTGTTATCATCCCT	23	58,9	145
				R2_10	GAATGAGCAATCACACATGACCT	23	58,9	
HaWOX11 (chr 1)	LOC110905286	XP_022006821.1	XM_022151129.2	F1_11	GCCCTGCAAACTCATGTGTGA	21	59,8	127
				R1_11	ATCTGTGGTGGTGAGTGGGA	20	59,3	
HaWOX12 (chr 16)	LOC110917354	XP_022017615.1	XM_022161923.2	F2_12	AGTGTTTATCAACGATGTGCCG	22	58,4	105
				R2_12	ACAGTCTGCCCGGATGAATC	20	59,3	

HaWOX13 (chr 11)	LOC110891845	XP_021995064.1	XM_022139372.2	F1_13	CACCTCACCGAGTTCTCTC	19	58,8	113
				R1_13	AACCCTTCTGTAAACCCCACA	21	57,9	
HaWOX14 (chr 17)	LOC110922699	XP_022022624.1	XM_022166932.2	F1_14	CACAAGGATTTTGCTTTCCCGAG	23	60,6	105
				R1_14	CAGTTGGCAGCATCCCACAT	20	59,3	
HaWOX15 iso_1 (chr 16)	LOC110915545	XP_022015954.1	XM_022160262.2	F1_15	GAATGCGGGTCAGATTTCGTA	21	57,9	83
				R1_15	CGGAGCCTCAATCTTTCCAAT	21	57,9	
HaWOX17 iso_1 (chr 15)	LOC110911924	XP_022012271.1	XM_022156579.2	F1_16	GAGTCACCCAAACCTCAGCA	20	59,3	82
				R1_16	CGACAGAACACGCATTTGGA	20	57,3	

Table S2. List of *WUSCHEL HOMEOBOX-like* (*HaWOX)* analyzed genes from sunflower (*Helianthus annuus* L.) and gene-specific primers used for quantitative Real Time PCR (qRT-PCR). In the first column, in brackets, are indicated the linkage groups (chr) of sunflower genome [44]. Information of publicly available repository of NCBI genes, transcripts and proteins are reported in the table. bp= base pair; Tm= temperature melting; Amp= amplificated nucleotides.

HaWOX	NCBI protein id	NCBI locus id	Chromosome number	Locus Start	Locus Stop	Strand	Length	Number of exons
HaWOX1	XP_022006965.1	LOC110905747	chr 14	111147922	111149801	+	293	3
HaWOX2	XP_022018228.1	LOC110918187	chr 16	200545038	200546829	+	343	3
HaWOX3	XP_021998889.1	LOC110895832	chr 12	98197915	98202980	+	347	4
HaWOX4	XP_022017513.1	LOC110917230	chr 16	36270578	36272102	+	242	3
HaWOX5	XP_022040761.1	LOC110943319	chr 1	67922367	67928543	-	241	2
HaWOX6	XP_022007144.1	LOC110906266	chr 14	167839771	167840766	-	210	2
HaWOX7	XP_021983563.1	LOC110879415	chr 9	165599317	165600805	+	239	3
HaWOX8	XP_022040174.1	LOC110942716	chr 5	12617328	12620462	-	254	3
HaWOX9	XP_022033204.1	LOC110934532	chr 4	111297211	111299307	-	186	2
HaWOX10	XP_021975839.1	LOC110871325	chr 8	87986765	87988719	+	214	2
HaWOX11	XP_022006821.1	LOC110905286	chr 1	66052674	66054993	+	182	2
HaWOX12	XP_022017615.1	LOC110917354	chr 16	146057100	146060383	-	389	5
HaWOX13	XP_021995064.1	LOC110891845	chr 11	125959177	125962848	-	389	5
HaWOX14	XP_022022624.1	LOC110922699	chr 17	134462267	134464881	-	389	4
HaWOX15	XP_022015954.1	LOC110915545	chr 16	203021901	203025077	+	247	4
HaWOX16	XP_022015955.1	LOC110915545	chr 16	203021901	203025077	+	215	4
HaWOX17	XP_022012271.1	LOC110911924	chr 15	82272148	82275053	+	249	3
HaWOX18	XP_022012272.1	LOC110911924	chr 15	82272148	82275053	+	243	3

Table S3. List of WOX proteins in *Helianthus annuus* genome.

Clade	HaWOX	Database	Signature accession	Signature description	Signature start location	Signature stop location
WUS	HaWOX1	PANTHER	PTHR45940:SF2	PROTEIN WUSCHEL	4	291
	HaWOX1	Pfam	PF00046	Homeodomain	28	88
	HaWOX2	PANTHER	PTHR45940:SF2	PROTEIN WUSCHEL	38	340
	HaWOX2	Pfam	PF00046	Homeodomain	69	129
	HaWOX3	PANTHER	PTHR45940:SF13	WUSCHEL-RELATED HOMEOBOX 1	66	347
	HaWOX3	Pfam	PF00046	Homeodomain	83	142
	HaWOX4	PANTHER	PTHR47716	-	8	238
	HaWOX4	Pfam	PF00046	Homeodomain	110	169
	HaWOX5	PANTHER	PTHR45940:SF6	WUSCHEL-RELATED HOMEOBOX 2	12	240
	HaWOX5	Pfam	PF00046	Homeodomain	27	85
	HaWOX6	PANTHER	PTHR45940:SF3	WUSCHEL-RELATED HOMEOBOX 7	30	210
	HaWOX6	Pfam	PF00046	Homeodomain	54	114
	HaWOX7	PANTHER	PTHR47716	-	6	239
	HaWOX7	Pfam	PF00046	Homeodomain	109	168
	HaWOX8	PANTHER	PTHR45940:SF6	WUSCHEL-RELATED HOMEOBOX 2	10	225
	HaWOX8	Pfam	PF00046	Homeodomain	26	85
	HaWOX9	PANTHER	PTHR45940:SF4	WUSCHEL-RELATED HOMEOBOX 3	17	178
	HaWOX9	Pfam	PF00046	Homeodomain	19	78
	HaWOX10	PANTHER	PTHR45940:SF4	WUSCHEL-RELATED HOMEOBOX 3	47	207
	HaWOX10	Pfam	PF00046	Homeodomain	50	109
	HaWOX11	PANTHER	PTHR45940:SF4	WUSCHEL-RELATED HOMEOBOX 3	11	177
	HaWOX11	Pfam	PF00046	Homeodomain	15	73
Intermediate	HaWOX12	PANTHER	PTHR47288:SF1	WUSCHEL-RELATED HOMEOBOX 9	1	376
	HaWOX12	Pfam	PF00046	Homeodomain	49	109
	HaWOX13	PANTHER	PTHR47288:SF1	WUSCHEL-RELATED HOMEOBOX 9	1	376
	HaWOX13	Pfam	PF00046	Homeodomain	58	118
	HaWOX14	PANTHER	PTHR47288:SF1	WUSCHEL-RELATED HOMEOBOX 9	1	375
	HaWOX14	Pfam	PF00046	Homeodomain	50	109
Ancient	HaWOX15	PANTHER	PTHR46777:SF4	WUSCHEL-RELATED HOMEOBOX 13	4	238

	HaWOX15	Pfam	PF00046	Homeodomain	72	132
	HaWOX16	PANTHER	PTHR46777:SF4	WUSCHEL-RELATED HOMEOBOX 13	4	204
	HaWOX16	Pfam	PF00046	Homeodomain	72	132
	HaWOX17	PANTHER	PTHR46777:SF4	WUSCHEL-RELATED HOMEOBOX 13	3	249
	HaWOX17	Pfam	PF00046	Homeodomain	79	139
	HaWOX18	PANTHER	PTHR46777:SF4	WUSCHEL-RELATED HOMEOBOX 13	3	243
	HaWOX18	Pfam	PF00046	Homeodomain	79	139

Table S4. List of Pfam and PANTHER accession for each HaWOX subdivided per clade.

A

AtWOX6	**TL**N**LFP**VR
HaWOX10	**TL**Q**LFP**TT
HaWOX11	**TL**Q**LFP**LT
HaWOX9	**TL**Q**LFP**LT
HaWOX3	**TL**E**LFP**VN
HaWOX6	**TL**Q**LFP**VN
AtWOX5	**TL**Q**LFP**VN
AtWUS	**TL**P**LFP**MH
HaWOX1	**TL**P**LFP**IH
HaWOX2	**TL**P**LFP**IP
AtWOX2	**TL**P**LFP**LQ
AtWOX3	**TL**E**LFP**IS
AtWOX1	**TL**E**LFP**LR
HaWOX8	**TL**D**LFP**MH
HaWOX7	**TL**E**LFP**LH
HaWOX4	**TL**E**LFP**LH
AtWOX4	**TL**E**LFP**LH
HaWOX5	**TL**D**LFP**LH
	●● ●●●

B

AtWUS	**LEL**R**L**
HaWOX1	**LEL**S**L**
HaWOX2	**LEL**S**L**
	●●● ●

Figure S1. Sequence analysis of WUS-box motif: TLXLFPXX (**A**) and EAR motif: LXLXL or LELXL (**B**) in WOX proteins from *H. annuus* (Ha) and *A. thaliana* (At). Bold characters and red dots evidence completely conserved amino acid residues.

```
HaWOX3      MWMMGYNEPHEDHMNINMVESYTTNGTRKLRPLVPRPPPPTCAATSSSSYNRFH----EH    56
HaWOX1      ------------------------------------------------------------    0
HaWOX2      MCH---------------L------------PLY-KPSLHTTFTHAPFPFSFFIFLFIQS    32


HaWOX3      L--------LPINHHLA--AEQNKREFNSQQMVVVSSRWNPTPEQLQTLEELY-RRGTRT    105
HaWOX1      ---------METQQQQADIQDFGNKNSNTYACRQSSTRWTPTSDQIRILKELYYNNGIRS    51
HaWOX2      LTSKQTNKQTTMDNNQPQDQDLGNKTNNSYLCRQSTTRWTPTSDQIRILKELYYNNGIRS    92
                         :::     : .::  *:      ::**.** :*:: *:*** ..* *:

HaWOX3      PSAEEIQHITAQLRRYGKIEGKNVFYWFQNHKARERQKRRRQLDNPAKELLHQ-PPFQLT    164
HaWOX1      PTADQIQRIAAQLRQYGKIEGKNVFYWFQNHKARERQKKRFTPTAPPPPSHFSDHIN---    108
HaWOX2      PTADQIQRIAARLRHYGKIEGKNVFYWFQNHKARERQKKRFTPPAPPPPPPPTLPPSNTT    152
             *:*::**:*:*:**:***********************:*     *

HaWOX3      TDINILNLDQSIQRKDSEVEETRNWAIPTLSEKSMLR------ERTKKAAADEGWRPAEQ    218
HaWOX1      ----------------HHLPNAAPMQIPSHHHHYHHQEPPHVYAHPHKLYTTHHI-----    147
HaWOX2      TTMLTSPFS------DHHHHHHQHMQIQSHHLPYFYN------NQPVKLHATHHISP---    197
                              .  .     * :       .       :  *  : .

HaWOX3      QQEEEEEGEEGNDQLLL-LHRRRKSIPARNATWSHMMQCLSSSSPTNHITLVNNNNNSNTT    277
HaWOX1      ------GVGSSSQGVMGVGCGYGSVAME----KSFRKCSI--SPPGESKATGGI-GRNIG    194
HaWOX2      ---SEGGGSSSSQAFLPVGCSYGAITME----KSFRDCSI--SP-GEYTVSGGE-IRNLG    246
                   *  ...* .: :     ::  .    . : .*    ** .. .  ..    *

HaWOX3      TTTTVTT------SKTTPSL---V-----VSSHHDDVDSYETLELFPVNRTVDHGREQ-P    322
HaWOX1      SRSRISVDSCSFFDTIKPKTYEMFENRDQDEEQGEPSTEIETLPLFPIHDG--------N    246
HaWOX2      SWVEVDS-----------SCLDKMKSENETEDGGELSTDIETLPLFPIPGGSTTTTASGS    295
             :   :             .    .      ..  :   . *** ***:

HaWOX3      EKCFLQTSDVT----------------------------LNPRQFIEFLPLKN    347
HaWOX1      HHDFFGMRTANLPLEQGTGGYYTGGSWYRSDERASLELSLNSYGYYN------    293
HaWOX2      QHDFFKMKEA-LSSEHSNGGLYTDVNWYHSDGRTSLELSLNSYGYYDSC----    343
             .: *:    .                             **   : :
```

Figure S2. Amino acid sequences of three WOX transcription factors of *Helianthus annuus* (HaWOX). The putative acidic motifs are highlighted in magenta.

MEME motifs	Consensus sequences
Red	ILERLYRQGLRTPTADQIQEITARLSQYGKIEGKNVFYWFQNHKARERQK
Pale blue	MFVKVMTDEQMEVLRKQIAIYATICZQLVDLHKSLTSNHDLAGVRLGNIY
Pale green	KFSGGTRWNPTPEQIQ
Purple	AVDDDNICFQNPNVGSVDQRAKKVEPVFPSDASSKP
Yellow	FEVAVGPFNVKEAFGDDAVLIDSSGQTVVTNEWGVTLQSLQHGAFYYLVR
Dark green	NNGESEVETLELFPLPPTN
Blue	GSMLANPRMDRIIGKMEVPGSYQSYMQGD
Fuchsia	MASSNRHWPSMFKSKPTYTTRQDHQWHS

Table S5. Amino acid motifs consensus sequences as retrieved by MEME for HaWOX family.

>HaWOX1 (XP_022006965.1)
METQQQQADIQDFGNKNSNTYACRQSSTRWTPTSDQIRILKELYYNNGIRSPTADQIQRIAAQLRQYGKI
EGKNVFYWFQNHKARERQKKRFTPTAPPPPSHFSDHINHHLPNAAPMQIPSHHHHYHHQEPPHVYAHPHK
LYTTHHIGVGSSSQGVMGVGCGYGSVAMEKSFRKCSISPPGESKATGGIGRNIGSRSRISVDSCSFFDTI
KPKTYEMFENRDQDEEQGEPSTEIETLPLFPIHDGNHHDFFGMRTANLPLEQGTGGYYTGGSWYRSDERA
SLELSLNSYGYYN

>HaWOX2 (XP_022018228.1)
MCHLPLYKPSLHTTFTHAPFPFSFFIFLFIQSLTSKQTNKQTTMDNNQPQDQDLGNKTNNSYLCRQSTTR
WTPTSDQIRILKELYYNNGIRSPTADQIQRIAARLRHYGKIEGKNVFYWFQNHKARERQKKRFTPPAPPP
PPPPTLPPSNTTTTMLTSPFSDHHHHHHQHMQIQSHHLPYFYNNQPVKLHATHHISPSEGGGSSSSQAFL
PVGCSYGAITMEKSFRDCSISPGEYTVSGGEIRNLGSWVEVDSSCLDKMKSENETEDGGELSTDIETLPL
FPIPGGSTTTTASGSQHDFFKMKEALSSEHSNGGLYTDVNWYHSDGRTSLELSLNSYGYYDSC

>HaWOX3 (XP_021998889.1)
MWMMGYNEPHEDHMNINMVESYTTNGTRKLRPLVPRPPPPTCAATSSSSYNRFHEHLLPINHHLAAEQNK
REFNSQQMVVVSSRWNPTPEQLQTLEELYRRGTRTPSAEEIQHITAQLRRYGKIEGKNVFYWFQNHKARE
RQKRRRQLDNPAKELLHQPPFQLTTDINILNLDQSIQRKDSEVEETRNWAIPTLSEKSMLRERTKKAAAD
EGWRPAEQQQEEEEGEEGNDQLLLLHRRRKSIPARNATWSHMMQCLSSSSPTNHITLVNNNNNSNTTTTT
TVTTSKTTPSLVVSSHHDDVDSYETLELFPVNRTVDHGREQPEKCFLQTSDVTLNPRQFIEFLPLKN

>HaWOX4 (XP_022017513.1)
MFLTIRGMKVHQFTRAGGFWGDDESLSCGLTLGLCKRFSPLNPKLTSSHSTETCVTMNDISGSNSSSSSH
VTAAFDLRSFIRPESCPTDQLVSSDHKKDSPQDETHPGGTRWNPTQEQIGILEMFYKGGMRTPNAQQIEQ
ITAKLSKYGKIEGKNVFYWFQNHKARERQKQKRNNLGLNHSPRSTPSSTMINSISLNPIGEVVESPYKKC
RSWSFECLEKDEEDDKTLELFPLHPEGRSRSS

>HaWOX5 (XP_022040761.1)
MEANSSKETIITVAGKETTTAAPTPCRWNPTKEQIDMLENLYKQGVRTPTADQIQEITTKLQTFGHIEGK
NVFYWFQNHKARQRQKQKQDHLSYFHQYYLHHNHHPAVPPGIYPVPHHPNVLYGQCYIPQRDIGFYNQYP
KMLLPSATTTKRRSPRTTTRTKASFGAGTIAGGNNMIKSKMVNGKNYNHQETLDLFPLHPTGILQQKEAN
ANNVNVCDSNACTSGPSVRSQDQHFFDFFSI

>HaWOX6 (XP_022007144.1)
MHQHVRCIFSVQKQQLITHKKTQRFRNLNMDEGMSGFGLKPSRSTSTGAAGSKCGRWNPTNEQVKVLTDL
FRSGLRTPSTDQIQKISSQLSFYGKIESKNVFYWFQNHKARERQKRRRVYVENDQNDVTHVTKQHLVEVS
ESGRVIETLQLFPVNTLAFSDQSEKTKVYTNDHECKENTSPYTTVYTCGTEMENNYQQRHPSLDLRLSFM

>HaWOX7 (XP_021983563.1)
MGLSSMKVHQFTRGGGFWRDDESSSSGLTLGAFKRMRPLNPKLTSSGSTETCLTMNNNISVSNSSSSSSH
VTAAFDLRSFIRPESSPIQLVSPDHKKDSPQVETHPGGTRWNPTQEQIGILEMLYRGGMRTPNAQQIEQI
TSQLSKYGKIEGKNVFYWFQNHKARERQKQKRNNLGLTHSPRSIPSSTMINSIPSNPRGEVVESPYKKCR
SWSFECLEKDEEEDDHKTLELFPLHPEGR

>HaWOX8 (XP_022040174.1)
MDGCSGTSEVPAMVVGGGAAGTPAASRWNPTKEQIDMLENLYRQGLRTPTADQIQEITGRLQTYGHIEGK
NVFYWFQNHKARQRQKEKQDHLSLFRQYNHHHRHLHQPPFVHIPPPPNVVYEPCYIQQHNVGFYVPQAVS
PNTTTSSSLNRRSPRSAKPSSAGFVLMHQPEIAVAVAGGSSSNIINIEQEDIATRKNNSRQETLDLFPMH
PTGILQEPVDTTSNGGCHASRACLIYVRILYVVDYVELCILLDD

>HaWOX9 (XP_022033204.1)

MTSVPVRAPTRGGGGGGGSRWCPTPEQVMMLEGMYRGGLKTPTATQIQQITARLSIYGKIQGKNVFYWFQ
NHKARDRQKLRKKLMVLYQQHRLYPTHHHPFLPLHQVAGVEDSSECKPSMNNWKVDIPSDSTCKLTCDCS
LMTMMTMDHYATTPYCTRLPPKTLQLFPLTTMDLKEADDLSTTSKP

>HaWOX10 (XP_021975839.1)
MQTEGDDNITKSPIPFINTPSLRDPLRSNLINYRKMSATRLSNSGSGGGSRWCPTPEQVMLLEGMYRGGL
KTPTASQIQQITARLSLYGKIQGKNVFYWFQNHKARDRQKVRKKLLAMYQHQRLLTSYNSPCYHPFPIQD
GAVVEDNISSSKKNMVNEWNVDDQTSCKKVMCDCSFMTVMMMESTSPYCTRVPPKTLQLFPTTPAQTLNP
SQPK

>HaWOX11 (XP_022006821.1)
MTSVPVPVTTSGGGRWCPTPEQVMLLEGMYRGGLKTPTATQIQQIVSRLSVYGKIQGKNVFYWFQNHKAR
DRQKLRKKLMALYQQHRLYPPTQDHPFLPLHPVGGVEDVSNNKPMMNNWKVDLPSEHQPCKLMCDCPLMT
MMLMEHHGRTTHSSRIPPKTLQLFPLTTTDLKQDDEPTTSKP

>HaWOX12 (XP_022017615.1)
MASSNRHWPSMFKSKPTYTTHQDHQWHSSATNRSSPYTSGCEERTPEPKPRWNPKPEQIRILESIFNSGL
VNPPRDEIRRIRARLQEYGQVGDANVFYWFQNRKSRSKHKNRHLQKPQSHQSRTDYAVTSKAVTTSSSSS
DDKSSSKSIEFFLNSPTMSVNQQPHTYLGAGGGSGSSSSNHQHHGEFFQEPFFSPVVQQKTQAPTASSFT
QGFCFSELGTMIDQGNQHGNDGQKIVSSSGMLLTDLMMNHQYGIPSKNCGAKGVEEDNIIKMLSHSTPPP
AQLAAPTLVVPPSTSTTIAPSTISDIQGVEEAAGTGKAIVFINDVPFEVAVGQFNVKEAFGGDAMLIDSS
GQTVLTNEWGVTLQSLEHGSFYYLVRSFTYDHAGTVDLI

>HaWOX13 (XP_021995064.1)
MASSNRHWPSMFKSKPTCNTRHHHQWHSNDSNTLLSSASPCHRSPYTSGCEERSPEPKPRWNPKPEQIRI
LESIFNSGMVNPPREEIRKIRAQLQEYGQVGDANVFYWFQNRKSRSKHKTRNLQKSQNHLSPTTTKVTTP
SSSSSSEKSYSKSIDFLLNSPTASVNQHFCGGGVGGGFHHHLTEFSQEQPFCFPAVQQPQPQVTDAVATT
TVGFTEGFCFSEFGNVVSHGHNQGNVIEAVESGSGMLLTDLLMMNQQSVIQKMNDSKCVDEKTVKASSYN
YTAPLPPTSAVPNPTTTTVASTICDIQGVVEPMPAGKSTVFINDVAFEVAVGPFNVREAFGDDAVLIHSY
GQPVVTNEWGVTLQSLQHGAFYYLVRSFSYGQSSTIDLI

>HaWOX14 (XP_022022624.1)
MASSNRHWPSMFKSKSTYTTPQDQQWQSHGSNHRSSPYTSGCDQRNPEPKPRWNPRPEQIGILESIFNSG
LVNPPRDEIRRIRARLQEYGQVGDANVFYWFQNRKSRSKYKNRQLQKSQNHQSSPLYGGIMAAATTSSSS
SSGKSSSKSVEFFLNSPTASVNQQPQACVGEGGGNSLHQHHGEFFQDSFLFPVVQQKPVAPSAASFAQGF
CFPEPGTMIDQVNHHMNDDHTVVSRCGMLPTDLMMNHQYGIQSKNSRGKDVEEENSIKMLSHNTTTTTTP
LSPLAVPTLIVPPAPTTIVPSTLNDHQGVEEAGRRKSIVFVNDVPYEVDVGPFNIKEAFGDNVVLIDSSG
QTVVTDEWGVTLQSLQHGAFYNLVRSSTYDHAGTVDNMF

>HaWOX15 (XP_022015954.1)
MDQDDRNMFVKVMTDEQMEVLRKQIAIYATICQQLVDLHKSLTSNHDLAGVRLGNIYSDPLTTSGCHKFS
GRQRWTPTPVQLQILERLFDQGNGTPSKQKIKEITSELSQHGQISETNVYNWFQNRRARSKRKQQVSTTN
NGESEVETEVESPKPHQNSASAVDESNICFQNSNVGSADQRAKRVEPVCPSDASSKPNAGQISYYGTMLA
NPRMDRIIGKIEAPGSYQSYMQGDHDRLSAKNQTRFS

>HaWOX16 (XP_022015955.1)
MDQDDRNMFVKVMTDEQMEVLRKQIAIYATICQQLVDLHKSLTSNHDLAGVRLGNIYSDPLTTSGCHKFS
GRQRWTPTPVQLQILERLFDQGNGTPSKQKIKEITSELSQHGQISETNVYNWFQNRRARSKRKQQVSTTN
NGESEVETEVESPKPHQNSASAVDESNICFQNSNVGSADQRAKRVEPVCPSDASSKPNAGQISYYGTMLA
NPNYM

>HaWOX17 (XP_022012271.1)
MEQDEGNNNNNNGGMFVKVMTDEQMEVLRKQIAIYATICEQLVDLHKSLTSHHDLAGVRLGNIYNDPLVT
SGGHKFSGRQRWTPTPVQLQILERLFEQGNGTPSKQKIKEITSELSQHGQISETNVYNWFQNRRARSKRK

QQVSAPNNNGESEVETEVESPKPQQNSTSTADDNMCFQNPNACSVDQRAKKVEPVFPSDASSKPAGNVGQ
MSYYGSMLSNPRMDRIIGKMEVPGSYQSYMQGDEYNMTG

>HaWOX18 (XP_022012272.1)
MEQDEGNNNNNNGGMFVKVMTDEQMEVLRKQIAIYATICEQLVDLHKSLTSHHDLAGVRLGNIYNDPLVT
SGGHKFSGRQRWTPTPVQLQILERLFEQGNGTPSKQKIKEITSELSQHGQISETNVYNWFQNRRARSKRK
QQVSAPNNNGESEVETEVESPKPQQNSTSTADDNMCFQNPNACSVDQRAKKVEPVFPSDASSKPAGNVGQ
MSYYGRMDRIIGKMEVPGSYQSYMQGDEYNMTG

Figure S3. Amino acidic sequences of the WOX transcription factor family of *Helianthus annuus* (HaWOX).The homeodomain HB is highlighted in yellow. The intron position is highlighted in red. Between brackets corresponding protein id from NCBI database.

Clade	HaWOX Pairwise comparison	dS	dN	dN/dS	Duplication event
WUS	HaWOX1 HaWOX2	1,1897	0,3176	0,267	WGD
	HaWOX4 HaWOX7	0,6233	0,0841	0,1348	WGD
	HaWOX5 HaWOX8	1,6011	0,4512	0,2818	WGD
	HaWOX1 HaWOX3	54,9613	1,0841	0,0197	
	HaWOX1 HaWOX4	52,5691	1,8074	0,0344	
	HaWOX1 HaWOX5	53,0618	0,9234	0,0174	
	HaWOX1 HaWOX6	58,1724	0,7374	0,0127	
	HaWOX1 HaWOX7	50,3862	1,6729	0,0332	
	HaWOX1 HaWOX8	17,6915	1,0243	0,0579	
	HaWOX1 HaWOX9	53,0522	0,7716	0,0145	
	HaWOX1 HaWOX10	10,9451	0,8025	0,0733	
	HaWOX1 HaWOX11	54,2451	0,9985	0,0184	
	HaWOX2 HaWOX3	49,1504	1,2753	0,0259	
	HaWOX2 HaWOX4	7,288	0,8818	0,121	
	HaWOX2 HaWOX5	52,8214	0,9827	0,0186	
	HaWOX2 HaWOX6	57,7555	0,8548	0,0148	
	HaWOX2 HaWOX7	44,883	0,7674	0,0171	
	HaWOX2 HaWOX8	57,7625	0,8548	0,0148	
	HaWOX2 HaWOX9	55,3682	0,8526	0,0154	
	HaWOX2 HaWOX10	50,0957	0,9502	0,019	
	HaWOX2 HaWOX11	4,3512	1,0234	0,2352	
	HaWOX3 HaWOX4	4,4944	0,8311	0,1849	
	HaWOX3 HaWOX5	54,7529	1,0277	0,0188	
	HaWOX3 HaWOX6	7,935	0,9848	0,1241	
	HaWOX3 HaWOX7	52,5517	0,8037	0,0153	
	HaWOX3 HaWOX8	50,6578	0,9206	0,0182	
	HaWOX3 HaWOX9	52,1847	0,675	0,0129	
	HaWOX3 HaWOX10	23,4122	0,8832	0,0377	
	HaWOX3 HaWOX11	27,0204	0,8451	0,0313	
	HaWOX4 HaWOX5	50,4919	0,6831	0,0135	

	HaWOX4 HaWOX6	26,4252	0,905	0,0342	
	HaWOX4 HaWOX8	26,333	0,905	0,0344	
	HaWOX4 HaWOX9	56,6917	0,5731	0,0101	
	HaWOX4 HaWOX10	5,8006	0,7784	0,1342	
	HaWOX4 HaWOX11	59,6387	0,6106	0,0102	
	HaWOX5 HaWOX6	55,9531	0,9401	0,0168	
	HaWOX5 HaWOX7	50,698	0,6674	0,0132	
	HaWOX5 HaWOX9	49,4327	0,797	0,0161	
	HaWOX5 HaWOX10	49,9498	0,8525	0,0171	
	HaWOX5 HaWOX11	5,433	0,8452	0,1556	
	HaWOX6 HaWOX7	6,2837	0,8397	0,1336	
	HaWOX6 HaWOX8	3,1971	0,8588	0,2686	
	HaWOX6 HaWOX9	59,6439	0,852	0,0143	
	HaWOX6 HaWOX10	53,7752	1,0242	0,019	
	HaWOX6 HaWOX11	4,9812	0,8558	0,1718	
	HaWOX7 HaWOX8	50,6977	0,6673	0,0132	
	HaWOX7 HaWOX9	49,4344	0,797	0,0161	
	HaWOX7 HaWOX10	49,9501	0,8525	0,0171	
	HaWOX7 HaWOX11	5,4331	0,8452	0,1556	
	HaWOX8 HaWOX9	3,1492	0,6735	0,2139	
	HaWOX8 HaWOX10	3,3278	0,8159	0,2452	
	HaWOX8 HaWOX11	3,4997	0,6508	0,186	
	HaWOX9 HaWOX10	1,264	0,335	0,2651	
	HaWOX9 HaWOX11	0,419	0,1461	0,3487	
	HaWOX10 HaWOX11	1,31	0,3162	0,2414	
INT	HaWOX12 HaWOX14	0,4869	0,148	0,3039	WGD
	HaWOX12 HaWOX13	1,0625	0,3124	0,294	
	HaWOX13 HaWOX14	1,0333	0,2947	0,2852	
ANT	HaWOX15 HaWOX17	0,7581	0,0954	0,125	WGD
	HaWOX16 HaWOX17	0,7643	0,1011	0,1323	
	HaWOX16 HaWOX18	0,8514	0,1081	0,1269	
	HaWOX15 HaWOX18	0,7872	0,1149	0,1459	

Table S6. Ratio between neutral mutation (dN) and purifying selection (dS) for each HaWOX combination divided per clade.

Reference

[44] Badouin, H.; Gouzy, J.; Murat, F.; Staton, S.E.; Cottret, L.;… Langlade, N.B. The sunflower genome provides insights into oil metabolism, flowering and Asterid evolution. *Nature* **2017**, *546*, 148-152.
